# Process Recovery after CaO Addition Due to Granule Formation in a CSTR Co-Digester—A Tool to Influence the Composition of the Microbial Community and Stabilize the Process?

**DOI:** 10.3390/microorganisms4010017

**Published:** 2016-03-17

**Authors:** Marietta Liebrich, Anne Kleyböcker, Monika Kasina, Rona Miethling-Graff, Andrea Kassahun, Hilke Würdemann

**Affiliations:** 1 GFZ German Research Centre for Geosciences, Section 5.3 Geomicrobiology, Telegrafenberg, 14473 Potsdam, Germany; liebri@gfz-potsdam.de (M.L.); anne.kleyboecker@gfz-potsdam.de (A.K.); kasina@gfz-potsdam.de (M.K.); rona.graff@web.de (R.M.-G.); 2 Institute of Geological Sciences, Jagiellonian University, 30-063 Krakow, Poland; 3 Dresden Groundwater Research Center e. V., Meranerstr. 10, 01217 Dresden, Germany; akassahun@dgfz.de; 4 Department of Engineering and Natural Sciences, University of Applied Sciences Merseburg, Eberhard-Leibnitz-Str. 2, 06217 Merseburg, Germany

**Keywords:** over-acidification, process recovery, phosphate accumulating organisms, granule formation

## Abstract

The composition, structure and function of granules formed during process recovery with calcium oxide in a laboratory-scale fermenter fed with sewage sludge and rapeseed oil were studied. In the course of over-acidification and successful process recovery, only minor changes were observed in the bacterial community of the digestate, while granules appeared during recovery. Fluorescence microscopic analysis of the granules showed a close spatial relationship between calcium and oil and/or long chain fatty acids. This finding further substantiated the hypothesis that calcium precipitated with carbon of organic origin and reduced the negative effects of overloading with oil. Furthermore, the enrichment of phosphate minerals in the granules was shown, and molecular biological analyses detected polyphosphate-accumulating organisms as well as methanogenic archaea in the core. Organisms related to *Methanoculleus receptaculi* were detected in the inner zones of a granule, whereas they were present in the digestate only after process recovery. This finding indicated more favorable microhabitats inside the granules that supported process recovery. Thus, the granule formation triggered by calcium oxide addition served as a tool to influence the composition of the microbial community and to stabilize the process after overloading with oil.

## 1. Introduction

Biogas plants are often run below their maximum loading rate to prevent process failure. To optimize plant operation, it is important to gain a better understanding of the microbial community composition and its behavior during stress conditions, such as an increase in the organic loading rate (OLR). Changes in the microbial composition during shock loads need to be investigated, including over-acidification and deacidification. These investigations will help to reduce the risk of process failure, which drastically decreases the profitability of biogas plants [1].

In a previous study, we examined the composition of the microbial community during organic overloading using sewage sludge and rapeseed oil as substrates [2]. We showed that the coexistence of hydrogenotrophic and acetoclastic methanogens increased the process stability and the capacity for higher OLRs. Furthermore, hydrogenotrophic methanogens related to *Methanospirillum hungatei* and *Methanoculleus receptaculi* became more dominant after overloading. For testing purposes, the process was operated with the same substrates and calcium oxide (CaO) as an additive at a high OLR of 9.5 kg volatile solids (VS)·m^−3^·d^−1^. Phosphate-accumulating organisms (PAOs) such as *Dechloromonas*- and *Rhodocyclales*-like bacteria were present and may have contributed to maintaining a stable the process [3]. In addition, Kleyböcker *et al.* [4] showed that phosphate and the ratio of volatile fatty acids (VFAs) to calcium had an early warning function in terms of over-acidification.

CaO and sodium hydroxide (NaOH) have been tested as countermeasures against over-acidification resulting from overloading with rapeseed oil [5]. Although both additives were able to raise the pH to a neutral range (pH ≥ 7.0), rapid and sustainable recovery of the process was achieved only with CaO. The process recovery was found to be dependent on granule formation. Kleyböcker *et al.* [5] hypothesized that the granules contained salts of calcium and LCFAs as well as calcium and phosphate. The phosphate was very likely released by PAOs while they took up VFAs. Furthermore, the granules might have provided favorable microhabitats for methanogenic activity. Thus, the hypothesis for the process recovery comprised the following four mechanisms (1) the precipitation of LCFAs with calcium; (2) the precipitation of phosphate and calcium after phosphate release and acid uptake by PAOs; (3) the adsorption processes of fatty acids on the granules; and (4) acid degradation due to favorable conditions in the granules. In an experiment to increase the OLR at stable process conditions with the same substrates, we showed that LCFAs and calcium made up the outer layers of granules formed due to CaO addition. These granules offered interfaces that were covered with biofilms [6].

In this study, the composition of the microbial community in the granules and in the digestate is characterized. The investigated samples originated from a deacidification experiment with CaO (CaL) presented in Kleyböcker *et al.* [5]. The aim of this investigation is to further substantiate the mechanism of process recovery as well as to identify the key microbial players indicating process stability. Furthermore, microscopic methods were applied to gain a more profound understanding of granule composition and formation.

## 2. Experimental Section

### 2.1. Laboratory Scale Reactor and Experimental Setup

The reactor contained 23 L of sludge with a total solid content of 5% at stable process conditions. It was operated at 50 °C and mixed pneumatically. The hydraulic retention time (HRT) was 23 days. The process was overloaded with 9 kg VS·m^−3^·day^−1^ of rapeseed oil and 1 kg VS·m^−3^·day^−1^ of sewage sludge from a wastewater management plant applying enhanced biological phosphorus removal (EBPR). After the provoked over-acidification, only sewage sludge was fed. CaO was added during 8 days ranging between 220 and 880 mg·L^−1^·day^−1^ in order to stabilize the process. Once a day, one liter digestate was withdrawn and one liter of substrate was fed. For more details see [5].

### 2.2. Studied Material

**Figure 1:**
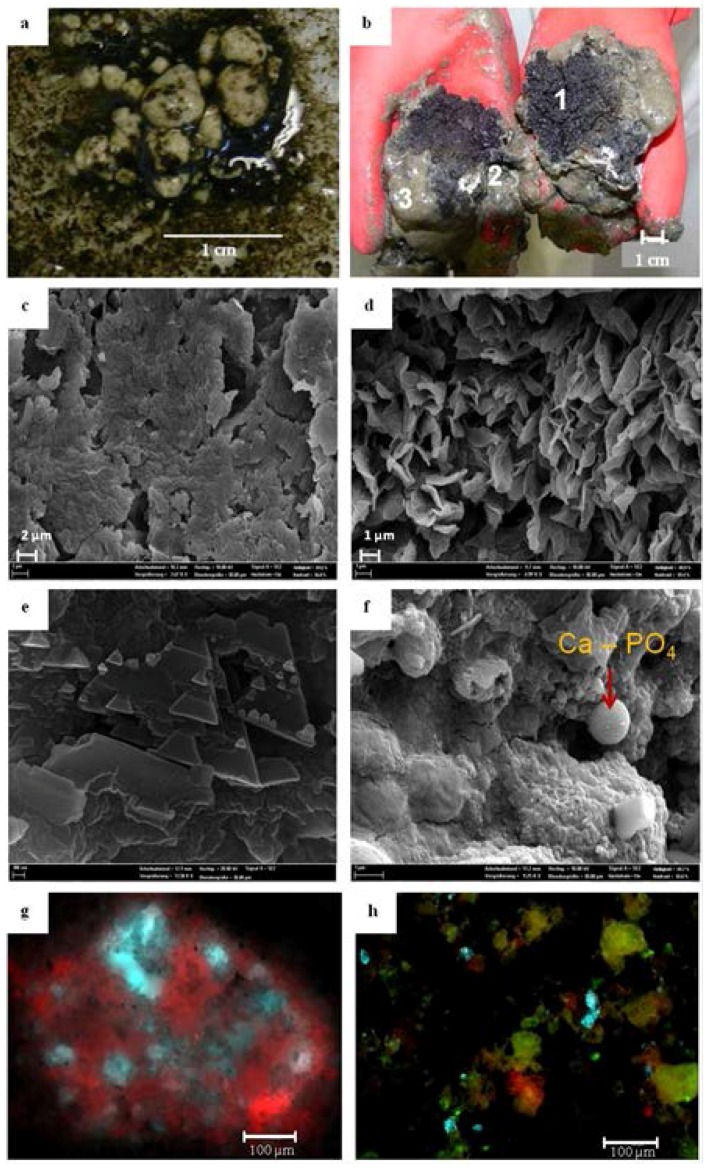
Granules: (**a**) Small white granules observed in the digestate during deacidification with CaO; and (**b**) broken large granule and three sampled zones marked with white numbers (Zone 1—black core; Zone 2—white/grey middle zone; and Zone 3—grey outer zone). SEM images of organic material of the granule (**c**) outer portion of the granule with a homogenous dense structure; and (**d**) inner portion of the granule with a porous structure in the leaf-like or teardrop-like shape. SEM images of phosphate minerals in the granule; (**e**) euhedral, trapezium-like Mg (Ca) phosphate minerals; and (**f**) rounded Ca phosphate minerals. Fluorescence microscopic images of granules (**g**,**h**) with close spatial relationships between calcium (red) and oil or LCFAs (cyan) and proteins (green).

Besides the digestate, small and large granules were investigated. Small white granules between 0.5 mm and 5 mm in size were frequently observed in the digestate after CaO additions (Figure 1a). In addition, after 12 days of operation, the reactor was opened, and few large granules with a maximum diameter of up to 7 cm were found. The granules were delicate with a porous structure. In a former study [6], we observed that the mineral composition of the small and large granules were quite similar. The large granule comprised different colored zones indicating different habitats. Therefore, it was chosen for examination of the structure, mineral composition, and microbial colonization of the zones, although it was thought to be too large for an efficient biogas production process.

### 2.3. Microscopic Analysis (SEM, Fluorescence and Light Microscopy)

To characterize the structure and spatial relationship between aggregate components and microbial colonization, granules were washed with distilled water and dried at 50 °C. The structures were analyzed with a scanning electron microscope (SEM) (Ultra 55 Plus, Carl Zeiss Microscopy GmbH, Jena, Germany).

The chemical composition of mineral components was determined quantitatively in spot analyses using an energy dispersive spectrometer (EDS). Samples were placed on special holders, coated with carbon, and examined at an acceleration voltage of 15 kV using secondary electrons (SE). Identification of components was provided by analytical software Thermo Noran NSS, Waltham, MA, USA.

For fluorescence microscopy, samples were stained with three different fluorescent dyes, *i.e.*, calcein (2,7-Bis(*N*,*N*-bis(carboxymethyl)aminomethyl)fluorescein), DiD (1,1′-dioctadecyl-3,3,3′,3′-tetramethylindodicarbocyanine) and DTAF (5-(4,6-dichlorotriazinyl)aminofluorescein). Calcein was used for detection of calcium (either free or bound), DiD for oil and LCFAs, and DTAF for proteins. The analyses were performed using an AF 6000 System (Leica Microsystems GmbH, Wetzlar, Germany; microscope control unit: CTR 6000, fluorescent lamp: EL 6000, camera: DFC 340 FX, LAS AF 2.3.0 software, Mannheim, Germany).

To prove the presence of volutin granules (intracytoplasmic polyphosphate storage), samples were stained with Neisser solutions according to Eikelboom and van Buijsen [7]. Samples of sewage sludge, digestate, and different zones of the large granules were stained using a staining kit from Merck (KGaA, Darmstadt, Germany). Analyses were performed using a Carl Zeiss Axio Imager.M2 (Carl Zeiss Microscopy GmbH, Jena, Germany; monochrome camera: AxioCam MRm, AxioVision 4.8.2 software, Göttingen, Germany).

### 2.4. Molecular Biological Methods

In this study, we used Polymerase Chain Reaction-Denaturing Gradient Gel Electrophoresis (PCR-DGGE) and quantitative real-time PCR (qPCR) to identify and quantify the dominant microorganisms. In a continuously stirred tank reactor, the normalized concentration decreases to 37% after one HRT due to wash out processes under ideal conditions [8]. Thus, inhibited organisms are washed out to 63% within one HRT. Because the duration of the experiment comprised 1.5 HRTs, the used methods were suitable to examine the key microbial players/dominant organisms.

Total DNA was extracted with buffer and silica beads according to a modified protocol [9]. For a 500 mg sample, extraction was carried out by adding 500 µL of extraction buffer (5 M GuSCN, 50 mM Tris HCl, pH 8, 25 mM NaOH, 1% SDS, 20 mM EDTA, 50 mM DTT), 250 µg of silica beads (0, 5–10 µm Sigma-Aldrich Chemie GmbH, Steinheim, Germany), and 500 µL of phenol/chloroform/isoamyl alcohol (25:24:1; Carl Roth GmbH + Co. KG, Karlsruhe, Germany) and mixing for 10 min on a Vortex-Genie 2 (Scientific Industries, Inc., New York, NY, USA). Afterwards, the samples were centrifuged for 5 min at 16,000× *g* and 4 °C. The supernatant was transferred to a new tube, mixed with the same volume of chloroform/isoamyl alcohol solution (24:1), and centrifuged at room temperature. Next, the samples were mixed with chloroform and centrifuged again. The supernatant containing the DNA was transferred to a new tube and was precipitated using a 5 M NaCl solution and isopropyl alcohol (C3H8O). To pelletize the DNA, the tube was centrifuged for 10 min at 14,000× *g*, followed by washing with 75% ethanol. Dried DNA was dissolved in 20 µL of water (nuclease-free, Thermo Fisher, Waltham, MA, USA), and stored at −20 °C until further analysis.

For genetic fingerprinting of the microbial community, PCR-DGGE analysis was carried out using specific primers for bacteria (341F-GC/907R; [10]), methanogenic archaea (348F-GC/786R, [11]) and PAOs (462F-GC/846R, [12]). All PCR reactions were conducted in a thermocycler (FlexCycler Analytik Jena, Jena, Germany) (95 °C for 3 min; 95 °C for 0.40 min; 54 °C (bacteria), 56.6 °C (archaea) for 0.45 min, 52 °C (PAO) for 0.40 min; 0.45 min (bacteria, archaea), 0.50 min (PAO) 72 °C; 72 °C for 10 min; 35 (40) cycles for bacteria (PAO and archaea)). All PCR reactions were carried out in a total volume of 50 µL containing 5 µL of 10 × buffer (GeneCraft, Ares Bioscience GmbH, Cologne, Germany), 3.5 µL of 50 × MgCl_2_ (GeneCraft), 6 µL of dNTP mix (2 mM each, Thermo Fisher), 3 µL of each primer (Eurogentec Deutschland GmbH, Cologne, Germany), 0.4 µL of Taq polymerase (5 U·µL^−1^, GeneCraft), 0.4 µL of BSA (20 mg·mL^−1^, Thermo Fisher), 27.3 µL of (bacteria) or 26.3 µL of (archaea, PAO) water (nuclease free, Thermo Fisher) and 1.5 µL (bacteria) or 2 µL of 1:10 diluted template. The PCR products were purified using a Fermentas GeneJET PCR Purification Kit (Thermo Fisher).

The DGGE analysis was performed according to Muyzer *et al.* [10] with equal concentrations of amplicons at 60 °C and 115 V for 17 h in a Biorad DCode System (Bio-Rad Laboratories GmbH, Munich, Germany). The gradient of the denaturants differed between the different primer sets for bacteria (35%–65% urea gradient with 6% acrylamide concentration), PAO (25%–60% urea gradient with 8% acrylamide concentration) and archaea (40%–60% urea gradient with 6% acrylamide concentration).

After electrophoresis, the polyacrylamide gel was silver stained, and the dominant bands were excised, re-amplified, and cleaned up using the Fermentas GeneJET PCR Purification Kit (Thermo Fisher). The PCR products were sequenced by GATC Biotech AG (Konstanz, Germany), and the sequences were compared to those in a publicly accessible database (NCBI GenBank) using BLAST (Basic Local Alignment Search Tool) [13]. The sequences in this study are available in the NCBI database under the GenBank accession numbers KU168212 till KU168243. Based on the DGGE profiles, a graphical representation of the bacterial community evenness was developed using the Pareto–Lorenz (PL) distribution curves [14]. The band intensities were determined using GelQuant.NET software [15]. The band intensities for every DGGE lane were ranked (high to low), and the cumulative band intensities were used as the *y*-axis. The cumulative normalized number of bands was set as the *x*-axis. Evaluation of the curves was conducted by comparison with a vertical 20% *x*-axis line. The theoretical perfect evenness line was set as a 45° diagonal.

Relative quantifications of archaeal and bacterial 16S rDNA genes were performed by qPCR with different primer sets for bacteria (BAC338F/BAC805R; [16]), archaea (ARC787F/ARC1059R; [16]) and PAOs (462f/846r, [12]) by applying the StepOnePlus™ Real-Time PCR System (Applied Biosystems—Life Technologies, Carlsbad, CA, USA). The fluorescent dye SYBR Green 1 (Power SYBR^®^ Green PCR Master Mix, Applied Biosystems) was used to measure the DNA increase during the PCR. Amplifications were carried out in 20 µL reactions containing 10 µL of Power SYBR^®^ Green PCR Master Mix, 0.5 µL of each primer (10 mM), 0.5 µL of BSA (20 mg·mL^−1^), 7.5 µL of water (nuclease free) and 1 µL of DNA (concentration of 0.2 ng·µL^−1^). A relative quantification was conducted according to the delta-delta-CT method [17]. Thereby, the ΔΔCT value was calculated according to Equations (1) and (2), using the CT values of the amplified archaeal and rPAO 16S rDNA fragments as well as the amplified bacterial 16S rDNA. The relative quantifications were determined by calculating 2^−ΔΔCT^.

(1) ΔΔCT = (CT_granule(archaea)_ − CT_granule(bacteria)_) − (CT_digestate(archaea)_ − CT_digestate(bacteria)_)
(2) ΔΔCT = (CT_granule(rPAO)_ − CT_granule(bacteria)_) − (CT_digestate(rPAO)_ − CT_digestate(bacteria)_)

## 3. Results and Discussion

After a provoked over-acidification, the process of biogas formation was recovered by the addition of CaO. To further substantiate the hypothesis regarding the role of calcium, the granules and digestate from the deacidification experiment presented in Kleyböcker *et al.* [5] were investigated using microscopic and molecular biological methods. Ten days after the successful process stabilization and restart of the co-substrate addition, neither large nor small granules were observed in the digestate. The degradation of granules was evidenced by both the increasing calcium concentrations and a methane yield above 3.0 m^3^·(kg VS)^−1^, which was seven times higher than expected (referring to the OLR during process recovery).

To investigate the impact of the microorganisms on the engineered process system and to identify the key players indicating stable process conditions, we focused only on the dominant organisms having a high relative abundance. In fact, Lawson *et al.* [18] showed a high activity of rare taxa, but the observed temporal activity patterns could not be explained by the measured process parameters. Thus, the activity of the rare taxa did not significantly influence the process of the engineered system.

### 3.1. Structure and Composition of Granules

During the deacidification process with CaO, the structure of the digestate changed, and small white granules between 0.5 mm and 5 mm in size were frequently observed (Figure 1a). The SEM-EDS analysis revealed that the granules were primarily composed of carbon and calcium. At day 12, the fermenter was opened, and few large granules with a maximum diameter of up to 7 cm were found.

The large granules displayed a layered structure with a black core (Zone 1) surrounded by white (Zone 2) and dark grey (Zone 3) materials (Figure 1b). The large granules consisted predominantly of organic material (60% of the dry matter) and phosphates of iron, aluminum and calcium. The organic material that formed the surface of the granule (Zone 3) exhibited a homogenous dense structure, whereas the organic material present in the inner part (Zone 1) was porous and appeared in the shape of flakes or teardrops (Figure 1c,d). The mineral phase of the large granule was 10 wt% greater the mineral phase of the digestate and consisted primarily of phosphate minerals with various shapes (Figure 1e,f) ranging from prismatic and trapezium-like to rounded and shapeless. The trapezium-shaped phosphate minerals were likely of inorganic origin, whereas the rounded phosphate minerals indicated a microbiological origin [19]. The various zones of the granules differed in their content of phosphate minerals and exhibited a negative gradient from the inner (Zone 1) to the outer region (Zone 3). SEM-EDS analysis confirmed the highest phosphate content in the core. This result indicates that the phosphate minerals originated from the sludge or were formed during granulation and served as a nucleus for granules beneath the precipitates of calcium with LCFAs and microorganisms. The outer region (Zone 3) of the granules was characterized by a higher content of calcium and carbon. Iron oxides as well as quartz, clay minerals, and feldspar were detected only in small quantities. Fluorescence microscopic analysis showed close spatial relationships of calcium, oil and/or LCFAs and proteins in all zones of the granules (Figure 1g,h). Oil and/or LCFAs were observed frequently, indicating that the carbon detected with SEM-EDS analysis was mainly of organic origin. The accumulation of LCFA salts adsorbed on granules in UASB reactors has been previously described by Hwu *et al.* [20]. The proteins identified in our study might be derived from the extracellular polymeric substances (EPS) of settled microorganisms. The proteins were distributed rather heterogeneously on the surfaces but in close spatial relationships with the precipitates of calcium and LCFAs. SEM observations by Kasina *et al.* [6] in a similar experiment with the same sludge and co-substrate revealed biofilms on the surfaces and interfaces of granules. This finding suggests that biofilms were also likely present within the granules investigated in this experiment.

### 3.2. Microbial Community Structure of the Digestate

**Figure 2:**
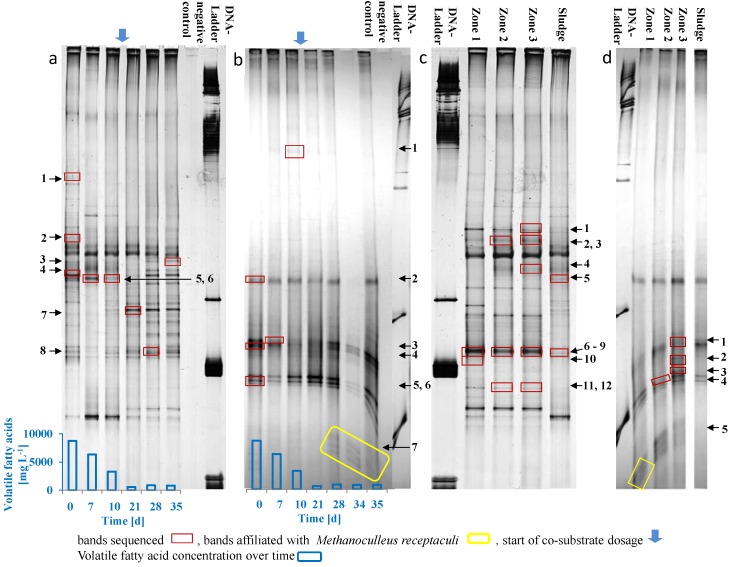
Genetic fingerprinting of digestate and granule samples of the different zones. Comparative DGGE analysis of PCR-amplified 16S rRNA gene fragments of: (**a**) *Bacteria* in the digestate; (**b**) *Archaea* in the digestate; (**c**) *Bacteria* in the different granule zones; and (**d**) *Archaea* in the different granule zones. The arrows indicate the bands that were sequenced.

Genetic fingerprinting with universal bacterial primers showed multifaceted and perturbance-resisting biocenosis in the digestate. Only slight changes were observed during over-acidification and process stabilization for the prevailing microorganisms (Figure 2a). The bacterial community in the digestate was dominated by the class *Clostridia* with six affiliated sequences, including relatives of the family *Clostridiaceae* and *Syntrophomonadaceae* (Table 1).

Both families are known to dominate LCFA-degrading microbial communities in the co-digestion of sludge and oil [21]. In particular, the genus *Syntrophomonas*, a member of the family *Syntrophomonadaceae*, was reported to degrade LCFA in anaerobic bioreactors [22]. Sequences assigned to the family *Syntrophomonadaceae* (band 9) were detected only after the VFA concentration decreased to less than 600 mg·L^−1^. This result is consistent with the observation of Regueiro *et al.* [23], who showed that the abundance of *Syntrophomonas* sp. decreased prior to a process failure. Thus, its appearance is interpreted as an indicator of favorable process conditions. Organisms related to the families *Chitinophagaceae* (band 1) and *Lachnospiraceae* (band 5, 6) were only detected within the first days of over-acidification. The relative abundance of an uncultured *Clostridia* member (band 4) changed during the experiment. The corresponding band disappeared after the first day and reappeared at day 21 after the VFA concentration decreased to less than 600 mg·L^−1^. Species of the genera *Clostridium* (band 3) and *Coprothermobacter* (band 8) were abundantly identified during the entire experiment. Representatives of *Clostridium ultunense* were described by Schnürer *et al.* [24] to produce mainly acetate by fermentation. The fermentation products of *Coprothermobacter* species are acetate, H_2_, and CO_2_ [25,26], among others.

**Figure 3:**
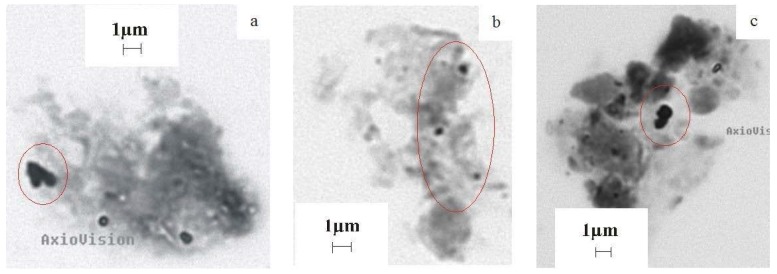
Light microscopy images from the three zones of the large granules containing polyphosphate storages highlighted in red ellipses: (**a**) Zone 1; (**b**) Zone 2; (**c**) Zone 3.

Candidatus *Accumulibacter phosphatis*-like organisms, relatives of *Rhodocyclus* sp. and species of the genus *Dechloromonas* were detected with primers specific for *Rhodocyclus*-related PAOs (rPAOs) in the digestate (Table 1). The DGGE profile of the rPAOs and the band intensities did not change during over-acidification and process recovery (profiles not shown). All detected rPAOs are well known from EBPR [27]. The PAOs release phosphate from their polyphosphate store when oxygen is depleted and VFAs accumulate in the liquor [28]. Accordingly, the phosphate concentration in the digestate reached its maximum at day six when the VFA concentration exceeded 7000 mg·L^−1^ and decreased with decreasing VFA concentrations [5]. The detection of rPAOs and the higher concentration of phosphate in the digestate during over-acidification as well as the occurrence of phosphate minerals with rounded shapes in the core of the granules indicate that the PAOs released phosphate and stored VFAs. Therefore, PAOs contributed to reducing VFAs in the granules. Correspondingly, microbial cells containing polyphosphate storage were detected by light microscopy in the granules (Figure 3a–c). Thus, the granule formation was initiated by the release of phosphate from PAOs and precipitated in the presence of calcium. This observation in turn supports our hypothesis regarding the mechanism of efficient process stabilization with CaO.

The archaeal community was dominated by members of the families *Methanomicrobiaceae* and *Methanosaetaceae* (Figure 2b, Table 1). Representatives of the family *Methanomicrobiaceae* (bands 5 and 6) and the genus *Methanosaeta* (band 4) were abundant throughout the over-acidification and recovery process and not washed out. *Methanosaeta* species are known to use acetate as energy source. In contrast, sequences related to *Methanoculleus receptaculi* (band 7) became more abundant in the digestate sampled after the deacidification process when the VFA concentration fell below 600 mg·L^−1^ and the biogas formation was recovered. *Methanoculleus receptaculi* is characterized as an obligate hydrogenotrophic organism and was first isolated from an oil field [29]. Strains of *Methanoculleus* have a high hydrogen affinity and can draw hydrogen to low levels [30]. Once the VFA concentration decreased to less than 600 mg·L^−1^, sequences related to *Methanoculleus receptaculi* became more abundant in the digestate, and therefore, their detection by genetic fingerprinting might serve as an indication of more favorable conditions for methanogenesis in the liquor.

### 3.3. Microbial Community Composition in the Granules

**Figure 4:**
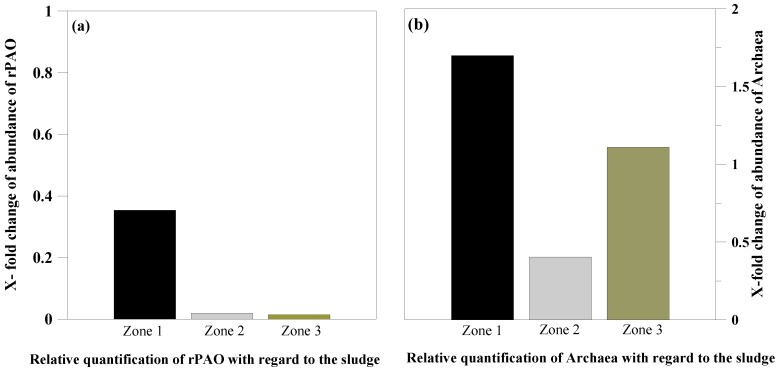
(**a**) Relative quantification of rPAO DNA compared to total bacterial DNA. Comparison of different granule zones to the digestate indicates a higher abundance of rPAOs in the core of the granule. Overall abundance was lower in the granule than in the sludge. (**b**) Relative quantification of archaeal DNA compared to total bacterial DNA. Comparison of different granule zones with the digestate indicates a higher abundance of archaea in all zones of the granule.

The bacterial and archaeal community in the granules showed only slight differences in abundance within the three zones of the granules (Figure 2c,d). Among rPAOs, relatives of Candidatus *Accumulibacter phosphatis*, *Rhodocyclus* sp. and species related to the genus *Dechloromonas* were detected using specific primers. The relative quantification of rPAOs showed the highest relative abundance in the core and the lowest in the outer region of the granule (Figure 4a). This result is consistent with the assumption that PAOs induced the granulation process.

However, the overall abundance of rPAOs was 65% lower in the core, 98% lower in the middle and 99% lower in the outer region of the granules compared to the digestate. Therefore, the rPAO distribution in the granules followed a similar trend to the phosphate mineral gradient. The lower relative abundance of the PAOs in the granules compared to the digestate might result from a higher growth rate of other microorganisms involved in the degradation of the organic substrate, while the PAOs only contributed to the decrease in VFA by uptake and intracellular storage. In another experiment with the same substrates focusing on the process performance at high organic loading rates, we detected an increase in pore size with granule age [6]. We assumed that this increase indicated the degradation of organic matter inside the granules. This finding is consistent with the relative increase in mineral content of the granules observed in this study. We interpreted the relative increase to suggest a more efficient organic degradation inside the granules. In accordance with the digestate, the bacterial community of the granule was dominated by the class *Clostridia*, with nine related sequences, and *Synergistia*, with one related sequence (Figure 2c, Table 2).

**Figure 5:**
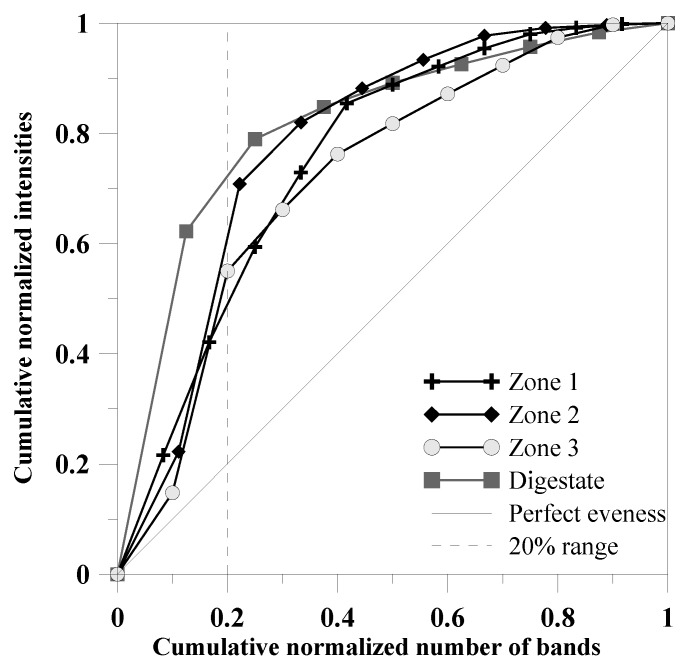
Pareto–Lorenz distribution curves based on the bacterial DGGE profiles of the different granule zones compared to the digestate for graphical representation of the bacterial community evenness. Perfect evenness is illustrated as a straight line. A dashed vertical line is plotted to evaluate the range of the Pareto value.

Sequences related to the order *Clostridiales* (band 1) revealed the highest abundance in the inner zone (Zone 1), as indicated by the strongest band intensity. In contrast, a lower abundance of this organism was detected in the outer zone (Zone 3) and in the sludge. Relatives of *Tepidimicrobium* sp. (bands 2, 3) and *Coprothermobacter* (bands 6–10) were detected in all granule zones as well as in the digestate and displayed the highest abundance in the granules. Furthermore, a *Clostridium*-like microorganism (band 4) was sequenced from samples of the middle and the outer zones of the granule but not from the core. Sequences related to the phylum *Synergistetes* were only detected in the granule (bands 11, 12). The family *Synergistaceae* includes the genus *Anaerobaculum.* Representatives of *Anaerobaculum* are thermophilic and convert malate to acetate, H_2_, and CO_2_. *Synergistaceae* were identified as “core” members of a mesophilic community found in municipal sewage sludge digesters [31]. Although the organisms of this family are frequently detected in anaerobic reactors, in this study, they were detected only in the granules, which indicates that they were not adapted to high organic acid concentrations. In contrast, relatives of the family *Lachnospiraceae* (band 5) were detected solely in the digestate, displaying a resistance against high acid concentrations. The Pareto–Lorenz distribution pattern of genetic fingerprinting analyses indicated a more even community within the granule (Figure 5). In the granule zones, 49% (Zone 1), 60% (Zone 2), and 55% (Zone 3) of the cumulative band intensities were derived from only 20% of the bands, whereas in the digestate, 71% of the cumulative band intensities were derived from only 20% of the bands. Both the granule zones and the digestate show several very intense bands, indicating higher relative abundances of only a few microorganisms that dominate the community. In particular, the dominating microorganisms in the over-acidified digestate were most probably well adapted to high acid concentrations.

Archaea (Figure 2c, Table 2) related to *Methanosaeta* (bands 1, 3), *Methanosarcinales* (bands 2, 4) and *Methanoculleus receptaculi* (band 5) were observed in all zones of the granules. Stronger band intensities indicated higher relative abundance of *Methanosaeta* (bands 1, 3) and *Methanoculleus receptaculi*-related organisms (band 5) in Zone 3. However, no difference was observed in the abundance of *Methanosarcinales*-related microorganisms (bands 2, 4). In contrast, relatives of *Methanoculleus receptaculi* were not observed in the over-acidified digestate. Detection of this organism in the inner zones of the granules during over-acidification is regarded as an indication of more favourable conditions inside the granules due to a lower concentration of VFA. Moreover, *Methanoculleus* is known to decrease the hydrogen partial pressure to low levels [30]. This point suggests that this organism lowered the hydrogen partial pressure in the granules, allowing the degradation of VFAs. Kleyböcker *et al.* [3] also found relatives of *Methanoculleus* and *Methanosarcina* dominating a high-performance biogas formation process stabilized by aggregate formation. Thus, the granules provide microhabitats with favorable conditions, even though the conditions in the digestate are unfavorable.

A relative quantification of archaeal DNA compared to the total bacterial DNA using qPCR showed a 70% higher abundance of archaea in the core (Zone 1) and an 11% higher abundance on the surface of the granules compared to the digestate, whereas in the middle zone, the abundance was 60% lower than in the digestate (Figure 4b). The higher content of archaea and the higher diversity of methanogens are regarded as evidence of better growth conditions and efficient methane production in the granules. The 60% lower abundance in the middle zone is likely attributable to the high oil content of this zone, which negatively affects microbial growth [32].

In summary, the analysis of the structure, mineral composition, and microbial colonization revealed that the granules provided a large surface for biofilm growth that facilitate the enrichment of syntrophic communities and a more even microbial community. Organisms related to the family *Clostridiaceae* (Figure 2c, bands 2–5), the genus *Coprothermobacter* (Figure 2c, bands 6–10) and the phylum *Synergistetes* (Figure 2c, bands 11, 12) as well as relatives of *Methanoculleus* (Figure 2d, band 5) and *Methanosarcinales* (Figure 2d, band 4) were detected in the granules. We assume that the consumption of hydrogen by hydrogenotrophic methanogens such as *Methanoculleus* reduced the hydrogen partial pressure within the granules. This process was promoted by LCFA precipitation with calcium, which significantly reduced the toxicity. These processes enabled the degradation of propionic acid within the granules by syntrophic bacteria. Therefore, propionic acid decreased rapidly after the addition of CaO despite a hydrogen partial pressure in the liquor that was far too high.

## 4. Conclusions

The addition of CaO after over-acidification with rapeseed oil stabilized the biogas process fed with sewage sludge and enabled continuous methane production in microhabitats of granules formed from Ca-LCFA salts and phosphate minerals of iron, aluminum and calcium. These granules provided sheltered niches for syntrophic communities. The presence of PAOs containing polyphosphate stores as well as the occurrence of phosphate minerals with rounded shapes indicated that PAOs released phosphate during the uptake of VFAs. Thus, they induced granule formation and contributed to the reduction of VFAs in the granules. *Methanoculleus receptaculi*-related organisms were detected in the inner zones of the granule, but only after process recovery in the digestate. The detection of these organisms in the granules clearly indicates the existence of favorable microhabitats and underlines their beneficial role in process recovery. Thus, induced granule formation serves as a tool to influence the microbial community and to stabilize the process. These conclusive observations regarding the microbial composition as well as the granule composition and formation confirm the hypothesis of Kleyböcker *et al.* [5].

## Figures and Tables

**Table 1:** Phylogenetic affiliation of partial bacterial, *Rhodocyclus*-related PAOs, and archaeal 16S rRNA gene sequences from DGGE profiles of the digestate during process recovery with CaO.

Domain	Band	Class	Family	Next Phylogenetic Relative (GenBank Accession Number)	Similarity (%)	Accession Number
**Bacteria**	**1**	*Sphingobacteria*	*Chitinobacteria*	Uncultured *Chitinophagaceae* bacterium (JF703505)	94	KU1682224
	**2**	*Clostridia*	*Clostridiaceae*	*Tepidimicrobium* sp. (KJ659943)	99	KU168225
	**3**	*Clostridia*	*Clostridiaceae*	Uncultured *Clostridium* sp. (KJ626485)	99	KU168226
	**4**	*Clostridia*		Uncultured *Clostridia* bacterium (KJ561290)	86	KU168241
	**5, 6**	*Clostridia*	*Lachnospiraceae*	Uncultured *Lachnospiraceae* bacterium (LC001683)	93, 97	KU168227, KU168228
	**7**	*Clostridia*	*Syntrophomonadaceae*	Uncultured bacterium (JF417905) Uncultured *Syntrophomonas* sp. (KC555203)	97 94	KU168229
	**8**	*Clostridia*	*Thermodesulfobiaceae*	Uncultured *Coprothermobacter* sp. (EU639297)	97	KU168230
**Archaea**	**1**	*Methanomicrobia*	*Methanosaetaceae*	Uncultured *Methanosaeta* sp. (KJ561304)	99	KU168212
	**2**	*Methanomicrobia*	*Methanomicrobiaceae*	Uncultured *Methanoculleus* sp. (KJ561301)	97	KU168213
	**3**	*Methanomicrobia*	*Methanosarcinaceae*	*Methanoculleus thermophilus* (KT368947, JF330114)	97	KU168214
	**4**	*Methanomicrobia*	*Methanomicrobiaceae*	Uncultured *Methanosaeta* sp. (KF692472)	98	KU168215
	**5, 6**	*Methanomicrobia*	*Methanomicrobiaceae*	*Methanomicrobiaceae* archaeon (GU129088)	92, 92	KU168216, KU168217
	**7**	*Methanomicrobia*	*Methanomicrobiaceae*	*Methanoculleus receptaculi* (NR_043961)	99	KU168218
**PAO**	**1**	*Beta-Proteobacteria*		Uncultured Candidatus *Accumulibacter* sp. (JQ726363)	98	KU136338
	**2**	*Beta-Proteobacteria*		Candidatus *Accumulibacter phosphatis* clade IIA (NR_074763)	99	KU136339
	**3, 4**	*Beta-Proteobacteria*	*Rhodocyclaceae*	*Dechloromonas* sp. (KF499998)	99, 98	KU136336, KU136337

**Table 2:** Phylogenetic affiliation of partial bacterial and archaeal 16S rRNA gene sequences detected by DGGE fingerprinting of the three different zones in large granules.

Domain	Band	Class	Family	Next Phylogenetic Relative (GenBank Accession Number)	Similarity (%)	Accession Number
**Bacteria**	**1**			Uncultured *Firmicutes* bacterium (JF681280)	87	KU168242
	**2, 3**	*Clostridia*	*Clostridiaceae*	*Tepidimicrobium* sp. (KJ659943)	99, 100	KU168231, KU168232
	**4**	*Clostridia*	*Clostridiaceae*	*Clostridium* sp. (AB093546)	99	KU168233
	**5**			Uncultured bacterium (HQ453302)	99	KU168234
		*Clostridia*	*Clostridiaceae*	Uncultured *Clostridiaceae* bacterium (AB089029)	98	
	**6, 7, 8**	*Clostridia*	*Thermodesulfobiaceae*	Uncultured *Coprothermobacter* sp. (KF208636)	100, 99, 100	KU168235– KU168237
	**9**	*Clostridia*	*Thermodesulfobiaceae*	Uncultured *Coprothermobacter* sp. (EU639297)	98	KU168238
	**10**	*Clostridia*	*Thermodesulfobiaceae*	*Coprothermobacter* sp. (AB162803)	96	KU168243
	**11**	*Synergistia*		*Synergistetes* bacterium (JF947039)	95	KU168239
	**12**	*Synergistia*		Uncultured *Synergistetes* bacterium (AB721098)	93	KU168240
**Archaea**	**1, 3**	*Methanomicrobia*	*Methanosaetaceae*	Uncultured *Methanosaeta* sp. (KJ561304)	99, 99	KU168219, KU168221
	**2**	*Methanomicrobia*	*Methanosarcinaceae*	Uncultured *Methanosarcinales* archaeon (LN896662)	93	KU168220
	**4**	*Methanomicrobia*	*Methanosarcinaceae*	Uncultured *Methanosarcinales* archaeon (LN796357)	94	KU168222
	**5**	*Methanomicrobia*	*Methanomicrobiaceae*	*Methanoculleus receptaculi* (NR_043961)	99	KU168223
**PAO**	**1**	*Beta-Proteobacteria*		Candidatus *Accumulibacter phosphatis* clade IIA (NR_074763)	99	KU136340
	**2**	*Beta-Proteobacteria*		Uncultured Candidatus *Accumulibacter* sp. (JQ726362)	99	KU136341
		*Beta-Proteobacteria*	*Rhodocyclaceae*	*Dechloromonas* sp. (KF499998)	99, 99	KU136342, KU136343
